# Reply to Dening, J. Comment on “Kolivas et al. A 6-Month mHealth Low-Carbohydrate Dietary Intervention Ameliorates Glycaemic and Cardiometabolic Risk Profile in People with Type 2 Diabetes. *Nutrients* 2025, *17*, 937”

**DOI:** 10.3390/nu18071115

**Published:** 2026-03-30

**Authors:** Despina Kolivas, George Moschonis

**Affiliations:** 1 School of Allied Health, Human Services & Sport, La Trobe University, Bundoora 3086, Australia; d.kolivas@latrobe.edu.au; 2 La Trobe Institute for Sustainable Agriculture & Food (LISAF), La Trobe University, Bundoora 3086, Australia

We appreciate the constructive feedback [1] and the opportunity to clarify some aspects of our manuscript. Regarding the comparison with the T2Diet study, we acknowledge that our original statement may have inadvertently emphasised between-group differences rather than within-group changes. We would like to thank you for highlighting this and agree that a clearer and more accurate representation would enhance the integrity of our manuscript. Upon revisiting the T2Diet publication, we confirm that the within-group reductions reported for the intervention group were indeed 0.94% for HbA1c, 4.36 kg for body weight, and 1.48 kg/m^2^ for BMI, all of which are statistically significant [2]. While it was not our intent to mislead the reader, this may have led to an underestimation in the degree of efficacy of the T2Diet protocol.

Regarding the second point about the use of the word “significant” with respect to changes in body weight status, we recognise that this phrasing has implied statistical significance for the full cohort, which was not the case. In our publications, while subgroup analyses revealed meaningful weight loss in a proportion of participants, the overall mean weight loss across the cohort was not statistically but clinically significant. While overall body weight did not change statistically significantly in the total sample, the majority of participants lost weight, and approximately 40% achieved clinically meaningful weight loss of ≥5%, which was associated with greater reductions in HbA1c [3].

With respect to the statistical methods, we acknowledge an oversight in our description of the timeframes for continuous outcome analysis. While the methods refer to baseline-to-3-month changes, we confirm that the same analytical approach was used to assess changes from baseline to 6 months that were presented in the paper [3].

Our data showed a consistent pattern in glycaemic control between the 3-month and 6-month time points, which is not unexpected given that participants maintained a similar level of adherence to the low-carbohydrate diet from 3 months onwards [3,4]. It is also important to note that further reductions or increases in HbA1c over time are not necessarily expected, as a dose–response relationship may not apply beyond the initial period of dietary change. The similarity in results over time, particularly regarding glycaemic control, aligns with findings from other studies showing that individuals following a low-carbohydrate dietary approach often maintain improvements over the longer term. Indeed, sustained glycaemic control has been reported in the literature for periods extending up to five years, regardless of the specific mode of dietary support or delivery [5,6,7,8,9,10].

Finally, we acknowledge the inherent limitations in dietary data collection and reporting [3,4]. As clearly noted in our discussion, the use of self-reported food records introduces variability and potential bias. However, our analysis showed consistent reductions in total energy and carbohydrate intake, which are aligned with glycaemic improvements, despite minimal weight change in the full sample. This is further supported by research demonstrating that improvements in glycaemic control can occur independently of substantial weight loss in low-carbohydrate interventions [11].

Future research will incorporate these learnings, specifically with regard to study design, statistical reporting, subgroup analyses, and dietary assessment methods. We reiterate our appreciation for the constructive comments, which have helped to clarify the results and interpretation of our paper and to inform our future research direction.
